# Genomic characterization of *Campylobacter* isolates in Huzhou, China

**DOI:** 10.1371/journal.pone.0289371

**Published:** 2023-08-17

**Authors:** Xiaofang Wu, Lei Ji, Yuehua Shen, Liping Chen, Deshun Xu, Fenfen Dong

**Affiliations:** Huzhou Center for Disease Control and Prevention, Huzhou, Zhejiang, China; Beni Suef University Faculty of Veterinary Medicine, EGYPT

## Abstract

*Campylobacter* is a major foodborne pathogen that causes outbreaks and sporadic gastrointestinal disease, creating a serious disease burden. *Campylobacter* strains isolated from diarrhea cases (n = 11) and raw poultry meat products (n = 2) in Huzhou, including 11 *Campylobacter jejuni* and 2 *Campylobacter coli* strains, were subjected to virulence gene, drug resistance gene, genetic correlation, antibiotic resistance, and multilocus sequence typing (MLST) analyses. The 13 *Campylobacter* isolates were divided into 12 sequence types (STs), one of which was a new ST. The isolated strains contain multiple virulence-related genes. Drug sensitivity analysis showed that the resistance rate of the 13 isolates to nalidixic acid, ciprofloxacin, and tetracycline was 92.3%. Genome sequencing indicated that all 11 strains of *C*. *jejuni* carried the tet(O) and blaOXA resistance genes, and 2 strains of *C*. *coli* carried multiple drug resistance genes. Phylogenetic analysis based on core-genome single-nucleotide polymorphisms indicated that the 11 *C*. *jejuni* isolates from diarrhea patients and food sources are not closely phylogenetically related.

## Introduction

*Campylobacter* is a zoonotic human pathogen that can cause diarrhea [1], and the main *Campylobacter* species causing infections in humans are *C*. *jejuni* and *C*. *coli* (accounting for more than 90%) [2]. The main clinical symptoms of *Campylobacter* infection are diarrhea and fever, and immune damage such as Guillain–Barre syndrome (GBS) can also occur [3]. *Campylobacter* is the most commonly reported foodborne pathogen causing outbreaks and sporadic gastrointestinal illness in developed countries and thus carries a significant disease burden [4], with up to 2.5 million cases in the United States each year [5]. *Campylobacter* causes more diarrhea than *Salmonella* or *Shigella* in developing countries [6, 7]. Sporadic diarrhea cases [8] and food-borne illnesses [9] caused by *Campylobacter*-contaminated foods have been frequently reported in China in recent years. Currently, the pathogenic mechanism of *Campylobacter* infection in humans is unclear [10]. *Campylobacter* mainly infects the body through adhesion, colonization, invasion, toxin production, and other mechanisms, leading to disease; during this process, a variety of virulence genes participate in the expression of virulence factors [11]. The flagellin gene flaA is closely related to the invasiveness and pathogenicity of bacteria, while the CadF gene encodes an outer membrane protein of *Campylobacter jejuni* that plays an important role in the adhesion and invasion process between *C*. *jejuni* and host cells [12]. The cdt gene cluster is composed of cdtA, cdtB, and cdtC in series, among which cdtB occurs widely and is correlated with cytolethal distending toxin titer [13].

The presence of various virulence genes is common in cases of campylobacteriosis. Drug resistance is a major problem affecting *Campylobacter* research and infection control. In 2013, fluoroquinolone-resistant *Campylobacter* and macrolide-resistant *Campylobacter* were listed as drug resistance threats affecting public health by the United States Centers for Disease Control and Prevention [14]. In China, drug resistance in *Campylobacter* has been reported; in particular, multi-drug resistant strains of *C*. *jejuni* have emerged as a public health concern [7].

Conventional methods for the isolation and identification of *Campylobacter* include enrichment culturing, selective isolation, and biochemical identification. The culture conditions for *Campylobacter* are harsh and cultivation time is long, which prevents the rapid diagnosis of food-borne diseases caused by *Campylobacter* infection. In recent years, whole-genome sequencing (WGS) has been widely employed in *Campylobacter* research [15]. Through systematic analysis of WGS data, the genomic characteristics and evolutionary relationships of various *Campylobacter* species can be determined. In this study, the whole genomes of 11 strains of *C*. *jejuni* and 2 strains of *Campylobacter coli* isolated from diarrhea patients and food sources were sequenced to obtain preliminary information about the genotypes and distribution characteristics of drug resistance genes, virulence genes, as well as the phylogenetic and evolutionary relationships among strains isolated in Huzhou. This study provides technical support for food safety risk assessment, prevention, and control of campylobacteriosis occurrence and development in the Huzhou area.

## Materials and methods

### Ethics statement

This study was approved by the human research ethics committee of the Huzhou Center for Disease Control and Prevention. The only human material used in this study is stool samples taken from patients for routine assessment. Oral informed consent was obtained from each participant.

### Bacterial isolates

A total of 13 strains of *Campylobacter* were isolated from diarrhea patients (stool samples) and raw poultry meat products collected at food markets during foodborne disease surveillance in 2022 across several regions of Huzhou. The 11 strains of *Campylobacter* obtained from diarrhea patients included 9 strains of *C*. *jejuni* and 2 strains of *C*. *coli*. Both strains of *Campylobacter* isolated from raw poultry meat products were *C*. *jejuni*. The 13 isolates were stored at −80°C in porcelain culture storage tubes (Qingdao Haibo, China).

### Antimicrobial susceptibility testing

The antimicrobial susceptibility of the 13 *Campylobacter* isolates was tested using the agar dilution method. The 11 antibacterial agents tested included the macrolide (erythromycin and azithromycin), quinolone and fluoroquinolone (nalididic acid and ciprofloxacin), aminoglycoside (gentamicin and streptomycin), chloramphenicol (chloramphenicol and florfenicol), tetracycline (tetracycline), ketolactone (telimycin), and lincosamide (clindamycin) classes of antibiotics. Antimicrobial susceptibility was interpreted as sensitive, intermediate, or resistant, with reference to the interpretation criteria described in the National Antimicrobial Resistance Monitoring System Drug Sensitivity Test Guidelines. The quality control strain for antibiotic resistance testing, *C*. *jejuni* (ATCC 33560), was obtained from Qingdao Zhongchuang, China.

### Genome sequencing

The whole-genome DNA libraries of 13 *Campylobacter* strains were constructed used the NGSmaster pathogen metagenomic one-stop library and kit. Next, the whole genome was sequenced using a NextSeq 550 sequencer (Illumina, USA).

### Multilocus sequence typing (MLST)

The sequences of seven housekeeping genes (aspA, glnA, gltA, glyA, pgm, tkt, and uncA) in the genome were used to determine the sequence type (ST) of *C*. *jejuni* and *C*. *coli* isolates through comparison with pubMLST (https://pubmlst.org/). If no ST is listed in the database matching the sequence of the strain, sequence alignment was conducted using BLAST to determine the new allele number of the housekeeping gene, which was applied as a new ST.

### Analysis of virulence genes

The virulence gene distribution was analyzed through WGS analysis of the strain. The genomes of *C*. *jejuni* and *C*. *coli* were submitted to The Virulence Factor Database (VFDB) to obtain virulence gene profiles. Pheatmap software was used to draw heatmaps based on the presence or absence of virulence genes.

### Analysis of drug resistance genes

The genomes of *C*. *jejuni* and *C*. *coli* were submitted to CARD (Comprehensive Antibiotic Resistance Database), and ABRicate software was used to predict and analyze the antibiotic resistance genes of the sequenced strains.

### Genetic correlation analysis

Snippy, Gubbins, and other software were used to sort the sequencing results and obtain the core-genome single-nucleotide polymorphisms (SNPs) of the strains. Sequence alignment and homology analysis of the 13 *Campylobacter* isolates were performed with Raxmal software.

## Results

### Genome sequencing

The average length of the genome sequences of 11 *C*. *jejuni* isolates was 172.15 kbp, and the GC content was 30%. The average length of the genomes of *C*. *coli* isolates was 168.97 kbp, and GC content was 31%. These lengths and GC contents are consistent with the genomic characteristics of *C*. *jejuni* and *C*. *coli* (Table 1).

**Table 1:** Genomic characteristics of 13 *Campylobacter* strains.

strain	species type	year	specimen origin	scaffold	full_len	GC	aspA	glnA	gltA	glyA	pgm	tkt	uncA	ST
CJS210483	C. jejuni	2021	patient stool	35	1785444	30.18%	24	2	2	2	10	3	1	464
CJS210739	C. jejuni	2021	patient stool	29	1662683	30.44%	9	53	2	10	11	3	3	305
CJS210740	C. jejuni	2021	patient stool	21	1687153	30.34%	24	2	2	72	22	406	6	4327
CJS210762	C. jejuni	2021	patient stool	14	1616462	30.39%	593	1	5	17	11	11	6	11775
CJS210763	C. jejuni	2021	patient stool	22	1669404	30.44%	2	1	5	10	608	1	5	6175
CJS210764	C. jejuni	2021	patient stool	34	1839562	30.21%	8	2	27	751	22	3	1	2133
CJS210768	C.jejuni	2021	patient stool	19	1603422	30.48%	4	7	10	4	42	51	1	583
CJS210770	C. jejuni	2021	patient stool	13	1648322	30.47%	55	21	2	71	11	37	3	2133
CJS22016	C. jejuni	2022	patient stool	34	1770540	30.22%	9	17	5	10	350	3	3	2274
CJS22073	C.jejuni	2022	raw chicken	17	1651525	30.35%	8	10	2	2	10	12	6	2988
CJS22074	C. jejuni	2022	raw chicken	29	1780830	30.15%	7	30	2	2	89	59	6	1213
ES210738	C.colon	2021	patient stool	20	1716051	31.36%	33	39	30	82	113	47	139	5511
ES210769	C.colon	2021	patient stool	20	1663437	31.44%	33	39	30	82	113	47	17	825

### MLST molecular typing

The 11 strains of *C*. *jejuni* were divided into 10 STs, with one strain (strain CJS210762) belonging to a new ST (ST, 11775). The two strains of *C*. *coli* were associated with two STs (Table 1).

### Analysis of virulence genes

**Fig 1:**
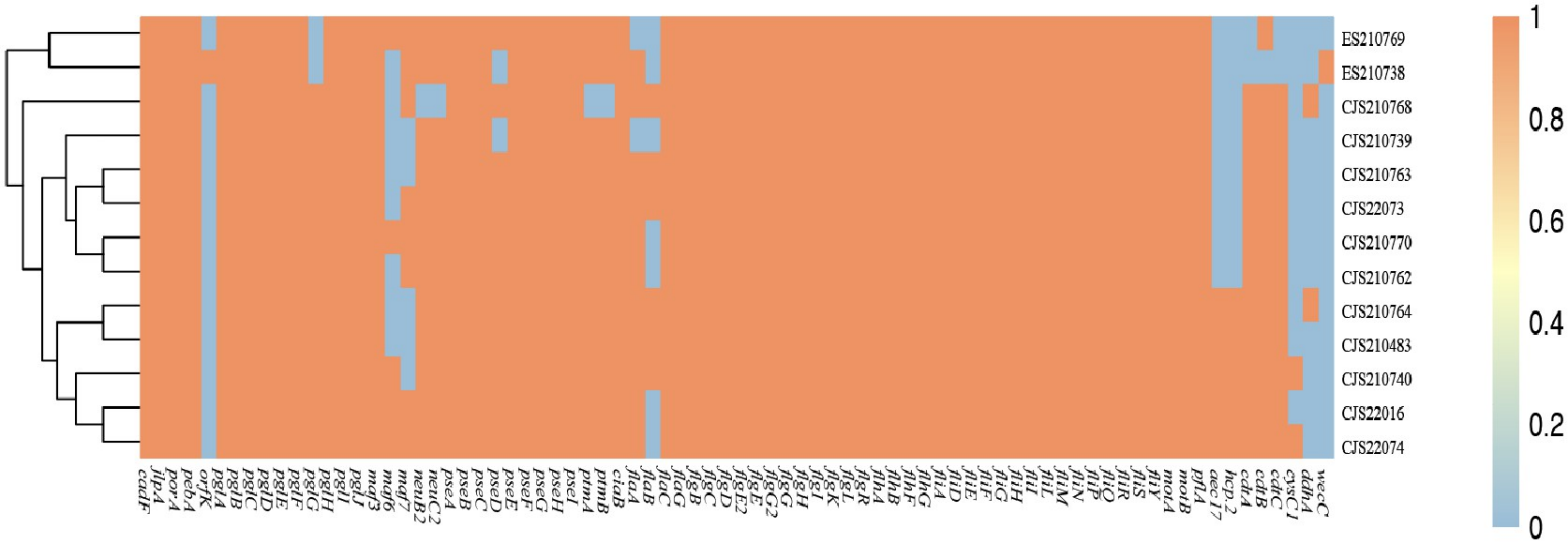
Virulence factors detected in the examined 13 *Campylobacter*i isolates in this study.

Analysis of virulence genes in the genomes of 13 *Campylobacter* strains showed that most strains carried more than 70 different virulence-related genes. No significant aggregation in the distribution of virulence genes was found among strains. Differences in the distribution of virulence genes were observed between *C*. *jejuni* and *C*. *coli*, as well as among strains of a given species. All 11 strains of *C*. *jejuni* contained three cytotoxic genes, cdtA, cdtB, and cdtC (Fig 1).

### Antimicrobial resistance and drug resistance gene analyses

The resistance rate of the 11 strains of *C*. *jejuni* to quinolones and tetracycline was 100%, and the resistance rate of the 2 strains of *C*. *coli* to tetracycline was 100%. Twelve of the 13 *Campylobacter* strains were resistant to three or more antibacterial drugs, with the remaining *C*. *coli* strain resistant to just one bacterial drug. Analysis of drug resistance genes indicated that the number of drug resistance genes was higher for *C*. *coli* than *C*. *jejuni*. All 11 strains of *C*. *jejuni* had tet(O) resistance genes and β-lactam antibiotic (blaOXA) resistance genes. Mutation analysis of the quinolone antibiotic resistance determinant region showed that C257T mutation occurred in the gyrA genes of 12 strains, all except CJS22016, which may be related to the quinolone antibiotic resistance of that strain (Table 2).

**Table 2:** Distribution of drug resistance genes in 13 *Campylobacter* strains.

strain	Strain type	specimen origin	Drug-resistant phenotype											Drug resistance gene							
			Macrolides		Quinolones		aminoglycosides		chloramphenicol		Tetracycline	Ketolactones	Lincoimides	Tetracycline	beta-lactam	Quinolones	chloramphenicol	aminoglycosides			
			ERY	AZI	NAL	CIP	GEN	STR	CHL	FLO	TET	TEL	CLI	*tet(O)*	*bla*_*OXA*_	*gyrA*	*catA13*	*aph(3’)-IIIa*	*aad9*	*aadE*	*AAC(6’)-Ie-APH(2’’)-Ia*
CJS210483	*C*.*jejuni*	patient stool	S	S	R	R	S	S	S	S	R	S	S	*+*	*+*	*+*	-	-	-	-	-
CJS210739	*C*.*jejuni*	patient stool	S	S	R	R	S	S	S	S	R	S	S	*+*	*+*	*+*	-	-	-	-	-
CJS210740	*C*.*jejuni*	patient stool	S	S	R	R	S	S	S	R	R	S	S	*+*	*+*	*+*	-	-	-	-	-
CJS210762	*C*.*jejuni*	patient stool	S	S	R	R	S	S	S	S	S	S	S	*+*	*+*	*+*	-	-	-	-	-
CJS210763	*C*.*jejuni*	patient stool	S	S	R	R	S	S	S	R	R	S	S	*+*	*+*	*+*	-	-	-	-	-
CJS210764	*C*.*jejuni*	patient stool	S	S	R	R	S	S	S	S	R	S	S	*+*	-	*+*	-	-	-	-	-
CJS210768	*C*.*jejuni*	patient stool	S	S	R	R	S	S	S	S	R	S	S	*+*	*+*	*+*	-	-	-	-	-
CJS210770	*C*.*jejuni*	patient stool	S	S	R	R	R	S	S	S	R	S	S	*+*	*+*	*+*	-	-	-	-	-
CJS22016	*C*.*jejuni*	patient stool	R	R	R	R	R	R	R	S	R	R	R	*+*	*+*	-	-	-	-	-	-
CJS22073	*C*.*jejuni*	raw chicken	S	S	R	R	S	S	S	R	R	S	S	*+*	*+*	*+*	-	-	-	-	-
CJS22074	*C*.*jejuni*	raw chicken	S	R	R	R	S	S	S	R	R	S	R	*+*	*+*	*+*	-	-	-	-	-
ES210738	*C*.*coli*	patient stool	S	S	S	S	S	S	S	S	R	S	S	-	*+*	*+*	*+*	*+*	*+*	*+*	*+*
ES210769	*C*.*coli*	patient stool	R	R	R	R	S	S	R	S	R	R	R	*+*	*+*	*+*	*+*	*+*	*+*	-	-

**Remarks:** "S" means sensitive, "R" means resistant; "+" has drug resistance gene, "-" has no drug resistance gene.

### Genetic correlation analysis

**Fig 2:**
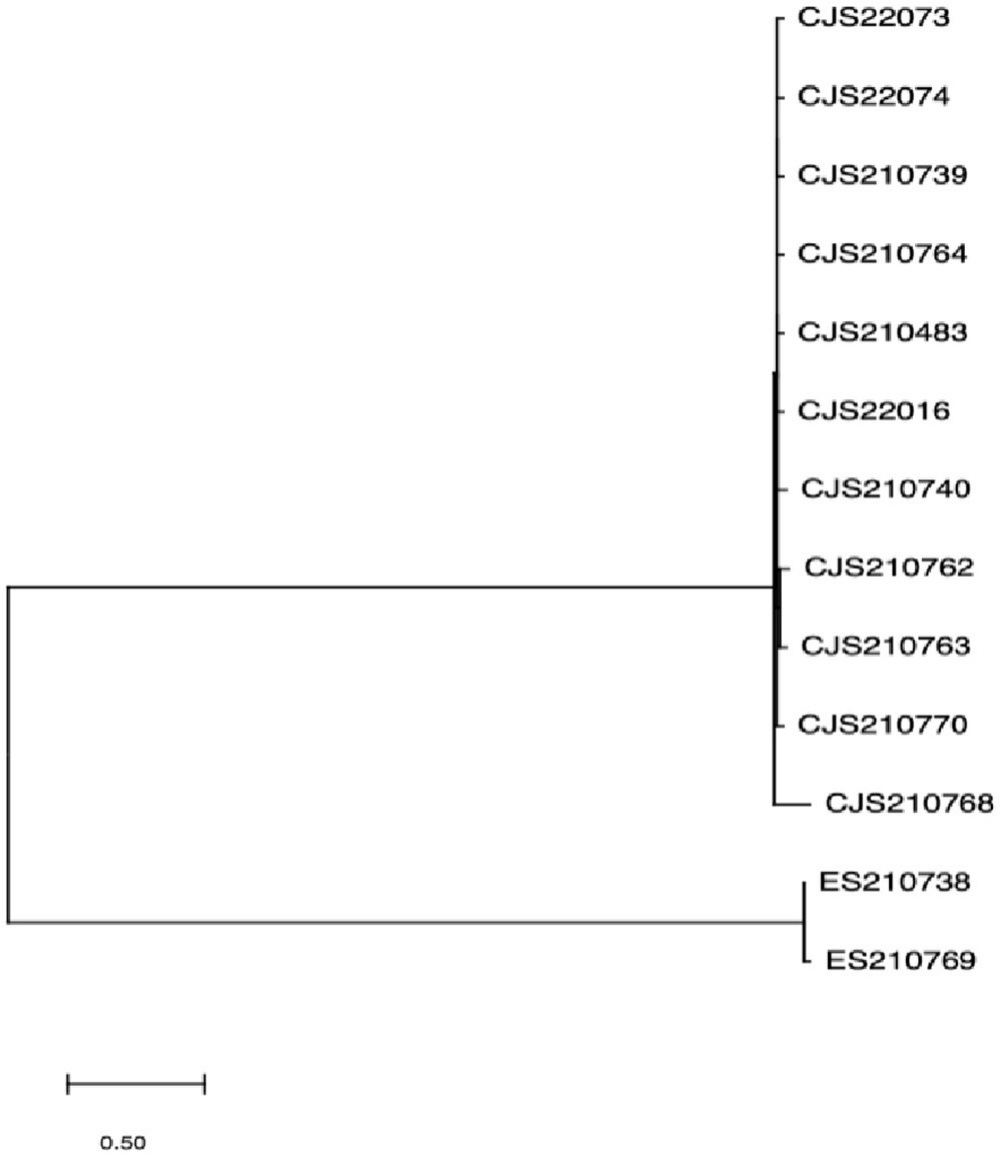
Phylogenetic tree based on cg-SNPs of 13 *Campylobacter* spp isolates.

**Fig 3:**
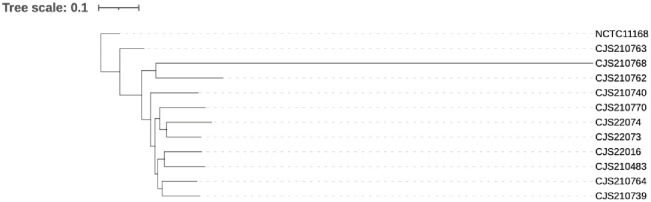
Phylogenetic tree based on cg-SNPs of 11 *C*. *jejuni* isolates, using the genome of strain NCTC 11168 as reference.

Core-genome SNP (cgSNP) clustering analysis based on the genome sequences of 13 isolates showed that *C*. *jejuni* and *C*. *coli* fall into two distinct groups (Fig 2). Using the genome sequence of NCTC 11168, the standard strain of *C*. *jejuni*, as the reference strain, cluster analysis of the genomes of 11 strains of *C*. *jejuni* indicated no apparent clustering of strains from different sources (Fig 3).

## Discussion

*Campylobacter* infection is considered a major cause of bacterial diarrhea in both developed and developing countries and is therefore an important public health problem [16]. Surveillance of *Campylobacter* in patients with diarrhea has been conducted in many provinces and cities in China, and *Campylobacter* has a higher detection rate than *Salmonella*, *Shigella*, and diarrhea-causing *Escherichia coli* [17–19]. With the improvement of living standards, food consumption is increasingly diversified and products are consumed in large quantities. The safety hazards associated with *Campylobacter* are also increasingly prominent. Therefore, clarifying the genetic characteristics of *Campylobacter* in this region provides technical information to support effective prevention of the ongoing *Campylobacter* epidemic.

WGS can be used to elucidate the molecular characteristics of virulence and drug resistance in various pathogens at the molecular level. In this study, WGS data for 9 strains of *C*. *jejuni* and 2 strains of *C*. *coli* isolated from stool samples of diarrhea patients as well as 2 strains of *C*. *jejuni* isolated from raw poultry meat samples were analyzed. The genomic characteristics of *C*. *jejuni* from these two sources were investigated, and no significant clustering was found. MLST is a classical bacterial molecular typing technique widely used to identify relationships among bacterial clones [20]. In this study, MLST analysis of 11 strains of *C*. *jejuni* and 2 strains of *C*. *coli* demonstrated the genetic diversity of their genotypes, in accordance with previous reports [17, 19, 21]. One *C*. *jejuni* strain was found to represent a new ST (ST11775). ST-464 is the most common ST in China [17, 19], and was detected in this study.

Bacterial adhesion, invasion, and flagellar activity are related to virulence and pathogenicity. Previous studies have confirmed that virulence genes such as flaA, flaB, CadF, and cdt are prevalent in *C*. *jejuni* and *C*. *coli* [22]. In this study, 13 *Campylobacter* isolates from two major sources carried more than 70 virulence-related genes, and the proportions of strains carrying the fibril binding protein gene cadF, invasion-related virulence gene ciaB, and flagellar gene flaA were very high. All 11 strains of *C*. *jejuni* contained three cytotoxic genes, cdtA, cdtB, and cdtC. This finding is consistent with Chinese and international reports [23, 24].

In recent years, fluoroquinolone and tetracycline antibiotics have been used as growth promoters in animal husbandry, resulting in a substantial increase in the proportion of drug-resistant bacteria [25]. The main mechanism of quinolone resistance is mutation of the GyrA gene in *C*. *jejuni*, while tetracycline resistance in *Campylobacter* is mainly due to the ribosome protective protein encoded by the tet(O) gene [26]. The results of drug sensitivity analysis showed that all 13 *Campylobacter* isolates were highly resistant to tetracycline and fluoroquinolone antibiotics. Drug resistance gene analysis indicated that all 11 strains of *C*. *jejuni* and 2 strains of *C*. *coli* had mutations of the tet(O) drug resistance gene and gyrA gene, with the 2 strains of *C*. *coli* carrying significantly more drug resistance genes than *C*. *jejuni*. However, this analysis also showed that the drug resistance genes were not fully expressed in the phenotypes of the isolated strains. The study of *Campylobacter* genetic characteristics has been suggested as an important means to elucidate the epidemic characteristics and evolutionary relationships of *Campylobacter*. In this study, based on cgSNP clustering analysis of core genes of the sequenced strains, *C*. *jejuni* and *C*. *coli* were found to cluster into two groups. The genomes of 11 *C*. *jejuni* isolates with different sources showed high diversity.

## Conclusion

We analyzed the antimicrobial resistance genes and conducted WGS of *Campylobacter* strains isolated from diarrhea cases and raw poultry meat in Huzhou. We assessed antibiotic resistance in *Campylobacter* in that region as well as the genetic characteristics of *Campylobacter* strains. This information will lay a foundation for the identification and pathogenicity analysis of sporadic and clustered outbreaks of *Campylobacter* infections in Huzhou.

## References

[1] Ekdahl K, Normann B, Andersson Y. Could flies explain the elusive epidemiology of *Campylobacteriosis*?[J]. BMC Infectious Diseases, 2005, 5(1):11. doi: 10.1186/1471-2334-5-11 15752427 PMC555947

[2] Fitzgerald C. Campylobacter[J]. Clin Lab Med, 2015, 35(2):289–298. 26004643 10.1016/j.cll.2015.03.001

[3] Winer J B. Guillain-Barré syndrome[J]. BMJ, 2008, 337:a671. doi: 10.1136/bmj.a671 18640954

[4] Hermans D, Pasmans F, Messens W, Martel A, Van Immerseel F, Rasschaert G, et al. Poultry as a host for the Zoonotic pathogen *Campylobacter Jejuni*[J].Vector Borne Zoonotic Dis, 2012, 12(2): 89–98. 22133236 10.1089/vbz.2011.0676

[5] Gaolin W, Baojun Y. Research progress of *Campylobacter jejuni* typing methods [J].Journal of Environmental Hygiene, 2006, 33(3):178–181.

[6] Coker AO, Isokpehi RD, Thomas BN, Amisu KO, Obi CL. Human *Campylobacteriosis* in developing countries[J]. Emerg Infect Dis, 2002, 8(3): 237–244. 11927019 10.3201/eid0803.010233 PMC2732465

[7] Acheson C, Allos BM. *Campylobacter jejuni* Infections: update on Emerging issues and trends[J].Clin Infect Dis, 2001, 32(8):1201–1206. 11283810 10.1086/319760

[8] Min L, Changyan J, Yanping M, Yongxiang D, Muhua Y, Maojun ZH, et al. Infection status of *Campylobacter* and its etiologic characteristics in diarrhea patients in Nanshan district, Shenzhen[J]. Disease surveillance, 2020.35(1):16–20.

[9] Xiaochun SH, Xiaohong ZH, Huiqun SH, Qinhong H, Xueqin ZH, Haoyue X. Epidemiology and etiology of an outbreak of *Campylobacter jejuni* foodborne disease[J].Chin J Sch Health, 2020, 41(11):1741–744.

[10] Wieczorek K, Wolkowicz T, Osek J. flaA-SVR based genetic diversity of multiresistant *Campylobacter jejuni* isolated from chickens and humans [J]. Front Microbiol, 2019, 10:1176. 31191494 10.3389/fmicb.2019.01176 PMC6546949

[11] Yaqi H, Jingyu X, Da SH, Zhenyu L, Darong CH. Research Advances on Virulence Factors and Pathogenic Mechanism of *Campylobacter jejuni*[J]. China Poultry, 2019, 41(22):46–51.

[12] Mead A J, Kharazi SH, Atkinson D, Macaulay I, Pecquet CH, Loughran S, et al. FLT3-ITDs instruct a myeloid differentiation and transformation bias in lymphomyeloid multipotent progenitors[J]. Cell Rep, 2013, (6):1766–1776. doi: 10.1016/j.celrep.2013.04.031 23727242 PMC3701326

[13] Bo L, Hui CH, Changyan H, Pengwei H. Study on the Differences of Virulence Genes and Molecular Typing in *Campylobacter jejuni* Isolates form Poultry Products and Diarrhea Patients in Shenzhen[J]. J Mod Lab Med, 2016, 31(5):107–109.

[14] CDC. Antibiotic resistance threats in the United Statess[EB/OL]. (2013-7-14)[2013-4-23]. http://www.cdc.gov/drugresistance/threat-report-2013/.

[15] Xi Y, Xinan J, Jinlin H. Research progress in *Campylobacter* genomic analysis[J]. Acta Microbiologica Sinica, 2021, 61(3):580–586.

[16] Maojun ZH, Yixin G, Ying L, Changyan J, Guilan ZH, Yunchang G, et al. Interpretation for the group standards of the Isolation and Identification of *Campylobacter jejuni* and *Campylobacter coli*[J]. Chin J Epidemiol, 2019, 40(9): 1052–1054. 10.3760/cma.j.issn.0254-6450.2019.09.006 31594144

[17] Ying L, Qiaoling J, Guilan ZH, Hongmei M, Yuanyuan W, Shuang ZH, et al. Surveillance and analysis of etiology characteristics of *Campylobacter* infection in adult diarrhea patients in Shunyi district of Beijing, 2016–2018[J]. Disease surveillance, 2020, 35(1):21–28.

[18] Yuanyuan W, Ying L, Shuang ZH, Yanchun ZH, Hongmei M, Maojun ZH. Infection status and drug resistance of *Campylobacter* in diarrhea patients in Shunyi district of Beijing, 2017[J]. Disease surveillance, 2018, 33(12):1048–1053.

[19] Leyi ZH, Huihuang L, Yuqin H, Lili W, Weiwei M, Yi L, et al. Research on distribution, drug resistance and molecular typing of foodborne *Campylobacter* in Wenzhou[J]. Chinese Journal of Zoonoses, 2020, 36(7):583–588.

[20] Huang J, Zhao Z, Zhang Q, Zhang SH, Chen M, Qiu H, et al. Phylogenetic analysis reveals distinct evolutionary trajectories of the fluoroquinolones resistant *Escherichia coli* ST1193 from Fu zhou, China[J]. Front Microbiol, 2021, 12:746995. 34803966 10.3389/fmicb.2021.746995 PMC8602892

[21] Zhang M, Gu Y, He L, L Ran L, Zhang J. Molecular typing and antimicrobial susceptibility profiles of *Campylobacter jejuni* isolates from north China[J]. J Med Microbiol, 2010, 59(10):1171‒1177. 20651041 10.1099/jmm.0.022418-0

[22] Maojun ZH, Yixin G, Lu R, Jianzhong ZH. Multi-PCR identification andvirulence genes detection of *Campylobacter jejuni* isolated from China[J]. Chin J Epidemiol, 2007, 28(4):377–380. 17850712

[23] Andrzejewska M, Klawe JJ, Szczepańska B, Spica D. Occurrence of virulence genes among *Campylobacter jejuni* and *Campylobacter coli* isolates from domestic animals and children[J].POI,J, Vet Sci, 2011,14(2): 207–211. 10.2478/v10181-011-0031-x 21721403

[24] Tingting S, Xue L, Weijie W, Tongzhu W, Zhenggang H, Tao X. Investigation of virulence gene of Camplobacter jejuni in the raw poultry meat in Liaoning Province[J]. Journal of Food Safety and Quality, 2022, 13(1): 318–323.

[25] Yanyan F, Yixin G, Li S, Yin L, Yongxiang D, Hao L, et al. Antibiotics susceptibility and genetic characteristice analysis for quinolone resistant *Campylobacter jejuni* isolated from China[J]. Chinese Journal of Zoonoses, 2018, 34(2):105–108.

[26] Maojun ZH, Jinzhong ZH. Status of resistance to *Campylobacter jejuni*[J]. Disease Surveillance, 2007, 22(12):793–796, 811.

